# Novel nitroxide-bile acid conjugates inform substrate requirements for human bile acid transporters

**DOI:** 10.1016/j.ejps.2022.106335

**Published:** 2022-11-17

**Authors:** Melissa Metry, Nathaniel D.A. Dirda, Jean-Pierre Raufman, James E. Polli, Joseph P. Y. Kao

**Affiliations:** a Department of Pharmaceutical Sciences, University of Maryland School of Pharmacy, 20 Penn Street, N623, Baltimore, MD 21201, United States; b Center for Biomedical Engineering and Technology, and Department of Physiology, University of Maryland School of Medicine, Baltimore, MD 21201, United States; c VA Maryland Healthcare System, Department of Medicine, Division of Gastroenterology & Hepatology, Department of Biochemistry and Molecular Biology, and the Marlene and Stewart Greenebaum Comprehensive Cancer Center, University of Maryland School of Medicine, Baltimore, MD 21201, United States

**Keywords:** Bile acids, Nitroxides, Transporters, Enterohepatic circulation

## Abstract

Transport of bile acids within the enterohepatic circulation from the liver to the intestines via the gallbladder and back to the liver via the portal vein plays a critical role in bile acid regulation and homeostasis. Deficiency of fibroblast growth factor 19 (FGF19), a hormone whose role is to suppress *de novo* hepatic bile acid synthesis to maintain homeostatic levels, results in bile acid diarrhea (BAD). FGF19 also modulates gallbladder motility so that bile acids are concentrated in the gallbladder until postprandial contraction. To assess bile acid transport and diagnose ailments like BAD that are associated with altered bile acid synthesis and transport, we created bile acid conjugates with nitroxide radicals. Because nitroxides are paramagnetic and can promote proton relaxation, we reasoned that these paramagnetic conjugates should act as contrast agents in *in vivo* magnetic resonance imaging (MRI). We tested substrate capability by assessing the inhibitory potential of these novel agents against taurocholate uptake by the apical sodium dependent bile acid transporter (ASBT) and the Na^+^/taurocholate cotransporting polypeptide (NTCP). Surprisingly, neither the paramagnetic compounds CA-Px-1 and CA-Px-2, nor their reduced forms, CA-Px-1H and CA-Px-2H, inhibited hASBT- or hNTCP-mediated taurocholate uptake. Therefore, the new conjugates cannot serve as contrast agents for MRI *in vivo*. However, our findings identify important structural constraints of transportable bile acid conjugates and suggest potential modifications to overcome these limitations.

## 1. Introduction

Bile acids are synthesized in the liver from cholesterol and transported in a highly conservative and coordinated cycle from the liver to the small intestine via the gallbladder and back to the liver via the portal vein within the enterohepatic circulation. An overall bile acid pool size of 2 to 4 g is maintained in humans and a small loss (<0.6 g/day) through the colon as feces is offset by *de novo* hepatic synthesis (Dawson, 2010). Bile acids are excreted from the liver into bile via the bile salt export pump (*ABCB11*, BSEP) and stored at very high concentrations in the gallbladder. After a meal, the gallbladder contracts to empty bile acids via bile ducts into the duodenum to promote food absorption. Bile acids are passively absorbed across all regions of the small intestine or exclusively at the ileum by active transport. The ileal apical sodium-dependent bile acid transporter (*SLC10A2*, ASBT) is responsible for the reclamation of the majority of bile acids from the ileum. Bile acids in the portal circulation are then transported back to the liver for uptake into hepatocytes via Na^+^/taurocholate co-transporting polypeptide (*SLC10A1*, NTCP). ASBT and NTCP are key transporters for enterohepatic circulation of bile acids to maintain proper bile acid homeostasis.

In addition to cholesterol metabolism and lipid solubilization, bile acids are signaling molecules and metabolic regulators. Bile acids play important roles in glucose and lipid metabolism, energy expenditure, intestinal and colonic motility, gut microbiota composition, intestinal inflammation, liver regeneration, and hepatocarcinogenesis. Dysregulated bile acid homeostasis can result in diabetes, dyslipidemia, cardiovascular diseases, hepatic steatosis, and cholestasis (Chiang, 2013). Bile acids, bile acid derivatives, and bile acid sequestrants are used therapeutically for such ailments. An aliment caused by disrupted bile acid homeostasis, bile acid diarrhea (BAD), can be misdiagnosed as irritable bowel syndrome (IBS-D). BAD manifests as chronic, watery diarrhea without ileal disease. BAD has been associated with deficiency in fibroblast growth factor 19 (FGF19), a protein that regulates *de novo* hepatic bile acid synthesis in response to the amount of bile acids in the ileum. Without the negative feedback control, bile acids are excessively produced, exceed the uptake capacity of ASBT, and spill into the colon. Augmented bile acid levels in the colon induce diarrhea by stimulating electrolyte, water, and mucus secretion. FGF19 also controls gallbladder motility to concentrate bile acids in the gallbladder until its postprandial contraction (Cheng et al., 2018; Choi et al., 2006).

The use of bile acid derivatives to assess impaired bile acid transport, particularly in detecting BAD, has been previously characterized *in vitro* and *in vivo*. For example, the accumulation of ^19^F-labeled bile acid analogues in murine gallbladders was visualized using ^1^H/^19^F magnetic resonance imaging (MRI) (Felton et al., 2016; Metry et al., 2018; Raufman et al., 2019; Vivian et al., 2014a; Vivian et al., 2014b; Vivian et al., 2013). Despite the promise of this approach, clinical translation was impeded by the lack of MRI facilities possessing the costly hardware and software necessary to detect fluorine signals or the financial incentive for such facilities to make this costly investment (Metry et al., 2018; Raufman et al., 2019).

**Fig. 1.**
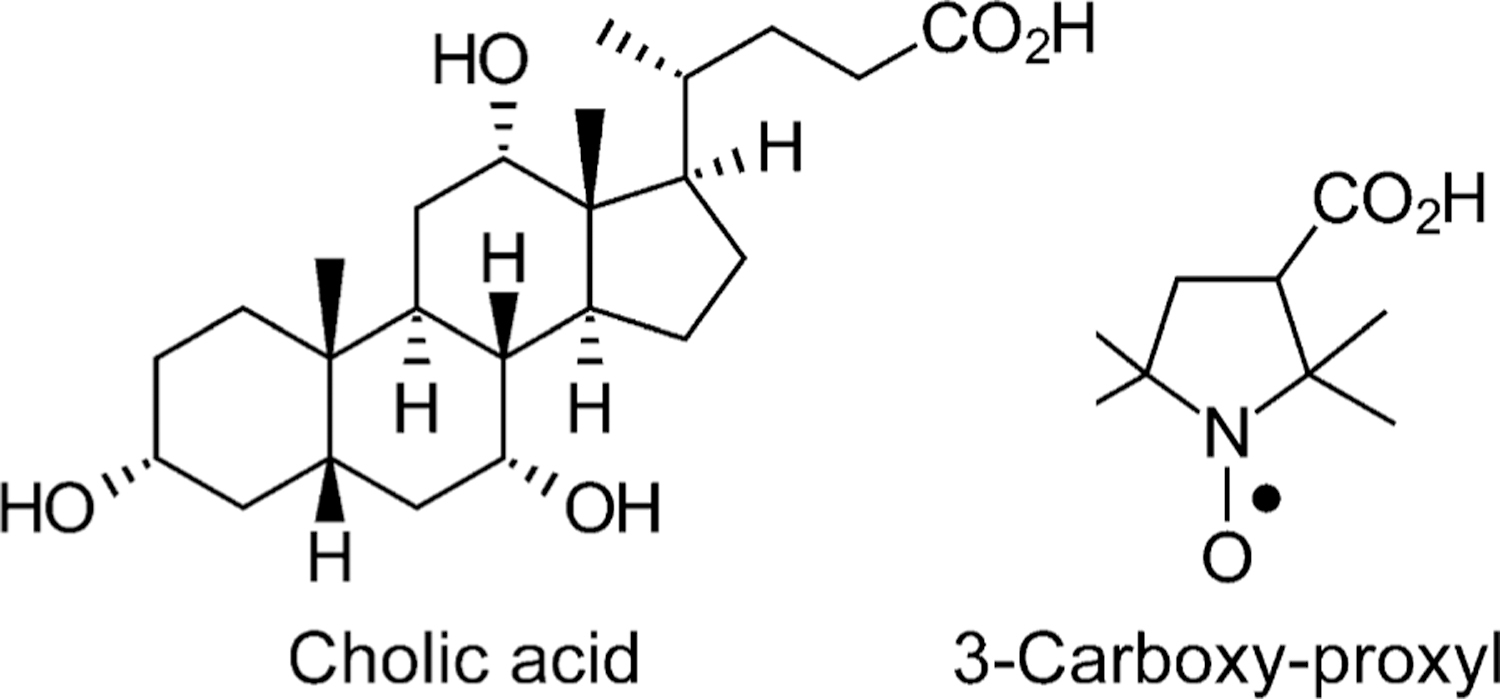
Cholic acid and 3-carboxy-proxyl (3-carboxy-2,2,5,5-tetramethylpyrrolidin-1-oxyl).

As an alternative to ^19^F-labeled bile acid analogue-MRI, we devised bile acids conjugated with molecules that can be visualized using conventional proton MRI. We conceived this novel ability to visualize enterohepatic circulation of bile acids had promise to diagnose BAD and other conditions resulting from altered bile acid transport. As reported here, we synthesized and tested two novel nitroxide-bile acid conjugates (NBACs). Nitroxides are stable, organic free radicals. Because the unpaired electrons on nitroxides can promote proton relaxation, they have been considered and studied as MRI contrast agents (Ehman et al., 1985; Grodd et al., 1987; Rajca et al., 2012). Nitroxides also have an excellent imaging and safety profile, and a long shelf-life. Thus, we designed NBACs to visualize bile acid accumulation and transport by conventional MRI without venipuncture, exposure to ionizing radiation, or additional infrastructure costs (i.e., no need for ^19^F-MRI hardware or software) – favorable features to facilitate clinical adoption of NBACs to detect BAD and other common gastrointestinal disorders resulting from altered bile acid synthesis or transport. The NBACs used in this study were prepared by conjugating cholic acid (CA) and 3-carboxy-2,2,5,5-tetramethyl-pyrrolidin-1-oxyl (“3-carboxyl-proxyl”) through different linkers (Fig. 1).

## 2. Materials and methods

### 2.1. Materials

Reagents and solvents for chemical synthesis were from commercial sources and were used as purchased, unless otherwise specified. [^3^H]-taurocholic acid was purchased from PerkinElmer (Waltham, MA). Taurocholate and glycocholic acid were obtained from Sigma Aldrich (St. Louis, MO), and cholic acid was from Alfa Aesar (Tewksbury, MA). Geneticin, trypsin, Dulbecco’s Modified Eagle Medium (DMEM), fetal bovine serum (FBS), nonessential amino acids, penicillin-streptomycin, and Turbofect^™^ transfection reagent were purchased from Invitrogen (Rockville, Maryland). Opti-MEM reduced serum medium and poly-*d*-lysine coated cultureware were obtained by Thermo Fisher Scientific (Waltham, MA). Plasmid PCMV5-NTCP was kindly provided by Dr. Peter Swaan. All other reagents and chemicals were of the highest purity commercially available.

### 2.2. Methods

#### 2.2.1. General methods for chemical synthesis

Products of chemical synthesis were purified on prepacked silica gel columns on a flash chromatography system (AKROS, Yamazen Science, Inc.). NMR spectra were recorded on a 400-MHz instrument (400 MR, Varian); samples were dissolved in either chloroform-*d*, dimethyl sulfoxide-*d*_6_, or D_2_O. High-resolution mass spectrometry (HRMS) was performed on an electrospray ionization (ESI) instrument (AccuTOF-CS, JEOL) at the Mass Spectrometry Facility in the Department of Chemistry and Biochemistry at the University of Maryland, College Park. All EPR spectra were recorded on an X-band spectrometer (EMXnano, Bruker); EPR samples were contained in 50-μl borosilicate capillary micropipettes (Drummond Scientific Company) whose ends were closed with sealing clay.

#### 2.2.2. Design rationale for the NBACs

We have previously reported the synthesis and testing of CA-sar-TFMA and CA-lys-TFMA, in which a fluorinated moiety was conjugated to cholic acid with either a sarcosine analogue or a lysine serving as linker. These conjugates were shown to undergo enterohepatic circulation, enabling their use as imaging agents in studying bile acid transport through ^19^F-MRI *in vivo* (Felton et al., 2016; Metry et al., 2018; Raufman et al., 2019; Vivian et al., 2014a; Vivian et al., 2014b; Vivian et al., 2013). CA-Px-1 and CA-Px-2, the NBACs prepared and tested in this study, are based structurally on CA-sar-TFMA and CA-lys-TFMA, respectively. Specifically, a nitroxide moiety (3-carboxyproxyl, Fig. 1) is conjugated to cholic acid through a sarcosine analogue to yield CA-Px-1, or through a lysine to yield CA-Px-2. Preparative details are described in the following sections.

#### 2.2.3. N-(2-aminoethyl)glycine methyl ester dihydrochloride, 1

To an oven-dried round-bottom flask *N*-(2-aminoethyl)glycine (1.00 g, 8.47 mmol) and dry methanol (35 ml) were added. The stirred suspension was cooled in an ice bath, and freshly distilled thionyl chloride was added dropwise to the cold mixture over 20 min. The resulting thick, white slurry was stirred for another 10 min at ice-bath temperature. Thereafter, the flask was fitted with a reflux condenser, and the stirred mixture was refluxed overnight. Rotary evaporation reduced the mixture to a nearly dry paste, which was dried on a vacuum line to give 1 as a white powder in quantitative yield. ^1^H-NMR (DMSO-*d*_6_), δ (ppm): 9.83 (s, broad, 2H), 8.43 (s, broad, 3H), 4.07 (s, 2H), 3.75 (s, 3H), 3.26 (d, *J* = 5.2 Hz), 3.22 (d, *J* = 5.2 Hz, 2H). HRMS(ESI^+^): [M+H]^+^, C_5_H_13_N_2_O_2_ requires 133.0977, found 133.0981.

#### 2.2.4. 3-{N-2-[(methoxycarbonylmethyl)amino]ethylcarbamoyl}−2,2,5,5,-tetramethylpyrrolidin-1-oxyl, 2

To an oven-dried round-bottom flask were added, in sequence, 1 dihydrochloride (0.5 g, 2.44 mmol), 3-carboxy-proxyl (0.454 g, 2.44 mmol), dry DMF (4 ml), and DIPEA (2.55 ml, 1.89 g, 14.6 mmol). After the suspension was stirred for 10 min, HBTU (0.925 g, 2.44 mmol) was added, and dry DMF (1 ml) was used to rinse down the flask walls. The now homogeneous reaction mixture was stirred under dry argon overnight. The solvent was removed under vacuum and the resulting viscous oil was partitioned between EtOAc (15 ml) and cold, saturated NaHCO_3_ (15 ml, adjusted to pH 9.5 with Na_2_CO_3_). The aqueous phase was extracted with EtOAc (2 × 15 ml), and the combined organic phase was dried over anhydrous Na_2_SO_4_, filtered, and reduced to a yellow oil by rotary evaporation, and further dried under vacuum. The crude material was purified by flash chromatography (EtOAc-MeOH gradient) to yield 2 as a yellow oil, weighing 0.36 g (49.2%). HRMS(ESI^+^): [M+H]^+^ C_14_H_27_N_3_O_4_ requires 301.20018, observed 301.2010.

#### 2.2.5. 3-{N-2-[(methoxycarbonylmethyl)cholylamino]ethylcarbamoyl}−2,2,5,5-tetramethylpyrrolidin-1-oxyl, 3

Compound 2 (0.360 g, 1.20 mmol) was added to a dry flask, followed by CA (0.979 g, 2.40 mmol), dry DMF (5 ml), and DIPEA (0.835 ml, 0.620 g, 4.79 mmol). The mixture was stirred under dry argon for 5 min and HBTU (0.909 g, 2.40 mmol) was added. The reaction mixture was stirred under dry argon overnight. The reaction mixture was partitioned between saturated NaHCO_3_ (20 ml) and EtOAc (25 ml). The aqueous phase was further extracted with EtOAc (4 × 25 ml). The combined organic phase was dried over anhydrous Na_2_SO_4_, filtered, and reduced by rotary evaporation to an oil, which was dried under vacuum to a thick gum. The crude material was purified by flash chromatography (EtOAc-MeOH gradient) to yield 3 as a yellow foam weighing 0.794 g (95.6%). HRMS(ESI^+^): [M+H]^+^ C_38_H_65_N_3_O_8_ requires 691.4772, observed 691.4778.

#### 2.2.6. 3-{N-2-[(carboxymethyl)cholylamino]ethylcarbamoyl}−2,2,5,5-tetramethylpyrrolidin-1-oxyl, 4 (CA-Px-1)

To a solution of compound 3 (0.1 g, 0.145 mmol) in a mixture of MeOH (2.6 ml) and water (0.75 ml) LiOH⋅H_2_O (30.4 mg, 0.724 mmol) was added. The reaction mixture was stirred for 100 min, at which point no starting material remained, as judged by TLC. MeOH was removed by rotary evaporation, the reaction mixture was partitioned between ice-cold 1 *M* glycine-HCl buffer (20 ml, pH 2) and EtOAc (20 ml). The aqueous phase was further extracted with EtOAc (3 × 15 ml). The combined organic phase was dried over anhydrous Na_2_SO_4_, filtered, and reduced to a solid yellow residue by rotary evaporation. The residue was purified by flash chromatography to yield CA-Px-1 as a yellow solid weighing 77.8 mg (79.4%). HRMS(ESI^+^): [M+H]^+^ C_37_H_63_N_3_O_8_ requires 677.4615, observed 677.4600.

#### 2.2.7. 3-{N-2-[(carboxymethyl)cholylamino]ethylcarbamoyl}−1-hydroxy-2,2,5,5-tetramethylpyrrolidine, 5 (CA-Px-1H)

CA-Px-1 (4) (68.0 mg, 0.100 mmol), 5% Pd/C (0.15 g), and MeOH (20 ml) were added to a hydrogenation bottle and shaken on a hydrogenator (Parr) under H_2_ (~30 psi) for 100 min. The mixture was filtered through celite into a flask containing 0.12 ml of 2 *M* HCl. The acidified filtrate was reduced by rotary evaporation and dried under vacuum to give CA-Px-1H (5) as a colorless solid in quantitative yield. ^1^H NMR (D_2_O), δ (ppm): 4.24 (s, 1H), 4.08 (s, 1H), 4.05 (s, 1H), 3.89 (s, 1H), 3.67–3.40 (m, 5H), 3.16–3.06 (m, 1H), 2.55–2.15 (m, 4H), 2.11–0.94 (m, 37H), 0.91 (s, 3H), 0.72 (s, 3H). HRMS(ESI^+^): [M+H]^+^ C_37_H_64_N_3_O_7_ requires 662.4745, observed 662.4149.

#### 2.2.8. N^2^-Cholyl-N^6^-[tert-butoxycarbonyl]-L-lysine methyl ester, 6

In an oven-dried flask, CA (1.377 g, 3.37 mmol) was dissolved in dry DMF (5 ml). To the stirred solution, HBTU (1.278 g, 3.37 mmol), DIPEA (2.35 ml, 1.74 g, 13.5 mmol), and the hydrochloride salt of *N*^6^-Boc-L-lysine methyl ester (1.00 g, 3.37 mmol) were added sequentially; solids adhering to the walls of the flask were washed down with 1 ml dry DMF. The flask was purged with dry argon and the mixture was stirred at room temperature (RT) for 21 h. Solvent was then removed under vacuum to leave a stiff gel, which was partitioned between water (25 ml) and EtOAc (35 ml). The aqueous phase was extracted once more with EtOAc (35 ml). The combined organic phase was washed once with cold 2 *M* KHSO_4_, dried over Na_2_SO_4_, filtered, reduced by rotary evaporation and dried under vacuum to give a white foam. The crude material was purified by flash chromatography (EtOAc-MeOH gradient) to yield 6 as a white foam weighing 2.030 g (92.6%). ^1^H-NMR (DMSO-*d*_6_) δ (ppm): 8.14 (d, *J =* 7.2 Hz, 1H), 6.78 (t, 1H), 4.33 (d, *J =* 4.8 Hz, 1H), 4.16 (m, 1H), 4.10 (d, *J =* 2.4 Hz, 1H), 4.01 (m, 1H), 3.78 (s, 1H), 3.59 (s, 4H), 3.16 (m, 1H), 2.88 (d, *J =* 6.4 Hz, 2H), 2.15 (m, 3H), 1.99 (m, 3H), 1.77 (m, 3H), 1.62 (m, 5H), 1.36 (s, 9H), 1.25 (m, 13H), 1.17 (m, 3H), 0.94 (d, *J =* 6.0 Hz, 3H), 0.81 (s, 3H), 0.58 (s, 3H). HRMS(ESI^+^): [M+H]^+^ C36H63N2O8 requires 651.4585, observed 651.4594.

#### 2.2.9. N^2^-Cholyl-L-lysine methyl ester, 7

To a stirred solution of compound 6 (0.75 g, 1.15 mmol) in dioxane (2 ml), ice-cold 6 *M* HCl (2 ml) was added over 1 min. After 45 min, TLC confirmed absence of the starting material. Volatile components were removed under vacuum overnight to give 7 as a clear lacquer (weighing 0.634 g, quantitative), which was shown by NMR spectroscopy to be essentially pure, and was used in the next reaction without further purification. ^1^H-NMR (DMSO-*d*_6_) δ (ppm): 8.95 (s, broad, 1H), 8.21 (d, *J =* 6.8 Hz, 1H), 8.06 (d, *J =* 6.8 Hz, 1H), 7.81 (s, 3H), 4.19 (m, 4H), 3.79 (s, 1H), 3.61 (m, 4H), 3.19 (m, 2H), 2.74 (m, 2H), 2.15 (m, 3H), 1.99 (m, 2H), 1.62 (m, 9H), 1.27 (m, 12H), 0.95 (d, *J =* 6.3 Hz, 3H), 0.81 (s, 3H), 0.59 (s, 3H).

#### 2.2.10. N^2^-Cholyl-N^6^-[2,2,5,5-tetramethyl-1-oxyl-3-pyrrolidinylcarbonyl]-L-lysine methyl ester, 8

Compound 7 (0.634 g, 1.15 mmol), 3-carboxyproxyl (0.215 g, 1.15 mmol), and HBTU (0.437 g, 1.15 mmol) were added to a flask, which was then purged with dry argon. Thereafter, DMA (3.5 ml) was added, followed by DIPEA. The mixture was stirred for 18 h under positive argon pressure. Volatiles were removed under vacuum to leave a thick oil, which was partitioned between water (20 ml) and EtOAc (20 ml). The aqueous phase was extracted once with EtOAc (20 ml). The combined organic phase was dried over anhydrous Na_2_SO_4_, filtered, reduced by rotary evaporation, and dried under vacuum to give a yellow foam. The crude material was purified by flash chromatography (EtOAc-MeOH gradient) to yield 8 as a yellow foam weighing 0.217 g (26.2%). HRMS (ESI^+^): [M+H]^+^ C_40_H_69_N_3_O_8_ requires 719.5085, observed 719.5068.

#### 2.2.11. N^2^-Cholyl-N^6^-[2,2,5,5-tetramethyl-1-oxyl-3-pyrrolidinylcarbonyl]-L-lysine, 9 (CA-Px-2)

LiOH (63.2 mg, 1.51 mmol) was added to a solution of compound 8 (0.217 g, 0.301 mmol) in a mixture of MeOH (5.4 ml) and water (1.6 ml). The mixture was stirred under argon atmosphere for 50 min and then most of the MeOH was removed by rotary evaporation. The remaining solution was chilled in an ice bath and ice-cold glycine-HCl (1 *M*, 20 ml) was added. The acidified mixture was extracted with EtOAc (3 × 20 ml). The combined organic extract was dried over anhydrous Na_2_SO_4_, filtered, and reduced to dryness by rotary evaporation. The crude material was purified by flash chromatography (EtOAc:MeOH) to yield 0.17 g (80%) of yellow solid. HRMS(ESI^+^): [M+H]^+^ C_39_H_67_N_3_O_8_ requires 705.49286, observed 705.4927.

#### 2.2.12. N^2^ -Cholyl-N^6^-[1-hydroxy-2,2,5,5-tetramethyl-3-pyrrolidinylcarbonyl]-L-lysine, 10 (CA-Px-2H)

CA-Px-2 (9) (0.0900 g, 0.128 mmol), 5% Pd/C (0.15 g), and MeOH (20 ml) were added to a hydrogenation bottle and shaken on a hydrogenator (Parr) under H_2_ (~30 psi) overnight. The mixture was filtered through celite into a flask containing 0.12 ml of 2 *M* HCl. The acidified filtrate was reduced by rotary evaporation and dried under vacuum to give CA-Px-2H (10) as a colorless solid weighing 94.0 mg (99.2%). ^1^H-NMR (D_2_O), δ (ppm): 4.31 (q, 1H), 4.05 (s, 1H), 3.88 (s, 1H), 3.67 (s, 1H), 3.56–3.44 (m, 1H), 3.31–3.08 (m, 3H), 2.4–2.2 (m, 4H), 2.05–1.20 (m, 42H), 0.98 (d, J = 5.9 Hz, 3H), 0.91 (s, 3H), 0.69 (s, 3H). HRMS (ESI^+^): [M+H]^+^ C_39_H_68_N_3_O_7_ requires 690.5058, observed 690.4432.

#### 2.2.13. Cell culture

Stably transfected hASBT-Madin-Darby canine kidney (MDCK; type-II) cells were cultured as previously described (Vivian et al., 2014a). Briefly, hASBT-MDCK cells were maintained in a humidified incubator at 37°C and 5% CO_2_ atmosphere in complete DMEM fortified with 10% (*v*/*v*) FBS, 50 units/ml penicillin, 50 μg/ml streptomycin, and geneticin (1 mg/ml) to maintain selection pressure. Cells were fed every 2 days and were passaged approximately every 4 days (after reaching 90% confluency). Cells were seeded in 24-well plates at a density of 5 × 10^5^ cells/well. One day after seeding and 12–15 h prior to assays described below, ASBT-MDCK cells were induced with 10 mM sodium butyrate.

Human embryonic kidney (HEK) 293T cells were cultured in a humidified incubator at 37°C and 5% CO_2_ atmosphere in complete DMEM fortified with 10% (*v*/*v*) FBS, 50 units/ml penicillin, 50 μg/ml streptomycin, and 1% (*v*/*v*) nonessential amino acids in poly-*d*-lysine coated cultureware. Cells were fed every 2 days and were passaged approximately every 4 days (after reaching 90% confluency). Cells were seeded in 24-well plates at a density of 5 × 10^5^ cells/well and transiently transfected 24 h later using pCMV5-hNTCP (accession: NM_003049.4), Opti-MEM® reduced serum medium, and Turbofect transfection reagent (0.5 μg plasmid DNA: 2 μl turbofect) according to the manufacturer’s directions. After 24 h post-transfection, hNTCP-HEK293T cells were used for the assays described below.

#### 2.2.14. Bile acid transport kinetics

The sodium (Na^+^)-dependent bile acid transport of taurocholic acid (TCA) was assessed by hASBT-MDCK cells and hNTCP-HEK293T cells. Cells were washed thrice with pre-warmed Hanks’ Balanced Salt Solution (HBSS) containing NaCl (8 g/l, 0.8%) or sodium-free solution with 137 mM TEA-Cl replacing NaCl. Cells were then incubated at 37°C for 5 or 10 min (periods of linear uptake for NTCP and ASBT, respectively) with 0–100 μM cold TCA spiked with 1 μCi/ml [^3^H]-TCA in HBSS or sodium-free buffer (SFB) donor solutions. Wells were quenched and then washed twice using ice cold sodium-free buffer (SFB). Cells were lysed with 300 μl ACN and left to evaporate at RT for 2–3 h. Lysates were resuspended in 1:1 ACN:H_2_O and centrifuged at 12,000*g* for 10 min. Supernatants were counted for radioactivity using a Tri-Carb® 2910 TR liquid scintillation counter (PerkinElmer, Waltham, MA). TCA uptake rates were determined as pmol/surface area (cm^2^)/min. Results were fitted to a straight line for SFB samples or a modified Michaelis–Menten equation for HBSS samples; no weighting was used, and regression was performed using Prism (GraphPad, San Diego, CA). This modified equation [Eq. (1)] accounts for passive permeability,

(1) $$V=\frac{V_{max}\left[S\right]}{K_{m}+\left[S\right]}+P_{pass}\left[S\right]$$

where V is TCA flux, [S] is TCA concentration and P_pass_ is passive TCA permeability. P_pass_ was determined on the same occasion by TCA uptake in SFB.

#### 2.2.15. Transporter inhibition potential

To test the ability of this assay to detect competition with TCA for transport via ASBT and NTCP, glycocholic acid (GCA) was used as a positive control inhibitor. Donor solutions consisted of 0–100 μM GCA in HBSS containing 2.5 μM TCA spiked with 1 μCi/ml [^3^H]-TCA. Competition of NBACs for transport via ASBT and NTCP were assessed using a similar assay where HBSS donor solutions consisted of increasing concentrations of NBAC with 2.5 μM cold TCA spiked with 1 μCi/ml [^3^H]-TCA. Inhibition rates were determined as pmol/cm^2^/min. Results were fitted to a modified Michaelis–Menten competitive inhibition equation; no weighting was used, and regression was performed using Prism (GraphPad, San Diego, CA). This modified equation [Eq. (2)] accounts for passive permeability,

(2) $$V=\frac{V_{max}\left[S\right]}{K_{m}\left(1+\frac{\left[I\right]}{K_{i}}\right)+\left[S\right]}+P_{pass}\left[S\right]$$

where V is TCA flux, [S] is TCA concentration and P_pass_ is passive TCA permeability. V_max_ and K_m_ were determined on the same occasion by TCA uptake in HBSS. P_pass_ was determined on the same occasion by TCA uptake in SFB.

#### 2.2.16. Statistical analysis

Data analysis was conducted using Prism (GraphPad, San Diego, CA). Results are expressed as the mean of three replicates ± standard error. Statistical comparisons were performed using the Student’s *t* test (assuming unequal variance). A critical p-value was used to determine statistical significance of TCA flux in the presence of NBACs compared to its absence.

## 3. Results

### 3.1. Synthesis of nitroxide-bile acid conjugates

**Scheme 1.**
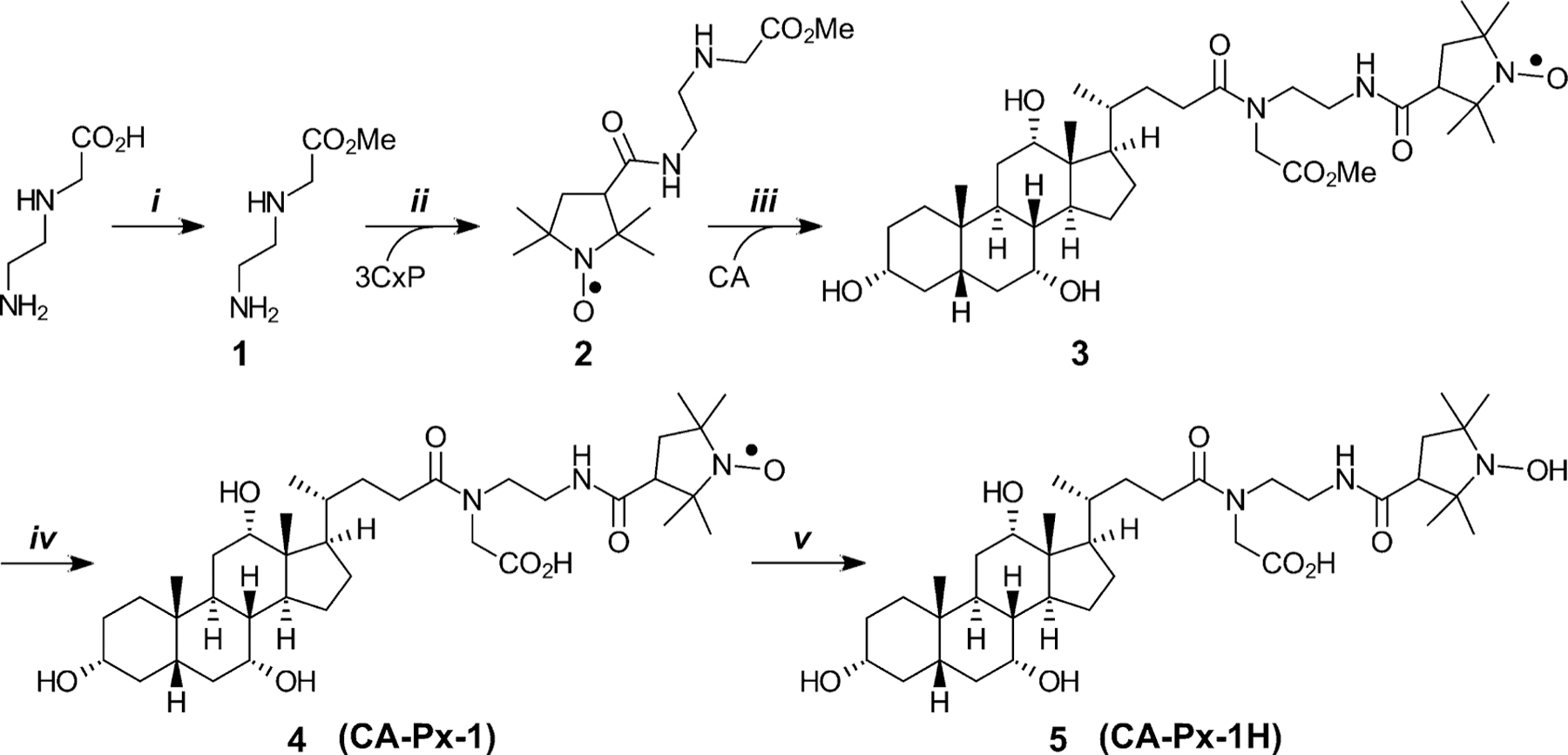
Synthesis of CA-Px-1 and its reduced form, CA-Px-1H. Reagents and conditions: *i*. SOCl_2_, methanol; *ii*. HBTU, *i*Pr_2_EtN, DMF; *iii*. HBTU, *i*Pr_2_EtN, DMF; *iv*. (a) LiOH⋅H_2_O, methanol/H_2_O, (b) glycine-HCl buffer, pH 2; *v*. (a) H_2_, Pd/C, methanol, (b) HCl. Abbreviations: 3Cx*p* = 3-carboxy-proxyl (3-carboxy-2,2,5,5-tetramethylpyrrolidin-1-oxyl); CA = cholic acid.

**Scheme 2.**
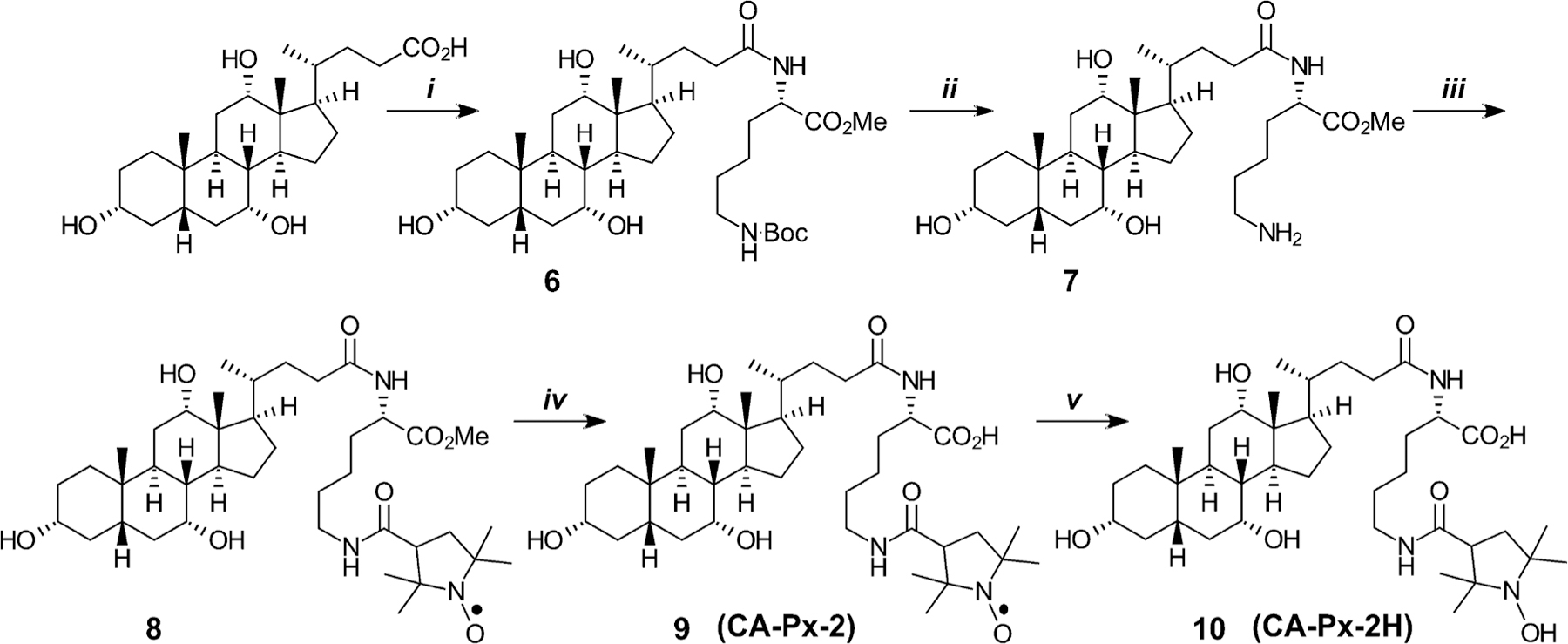
Synthesis of CA-Px-2 and its reduced form, CA-Px-2H. Reagents and conditions: *i*. H_2_N-Lys(Boc)-OMe, HBTU, *i*Pr_2_EtN, DMF; *ii*. 6 *M* HCl, EtOAc; *iii*. 3-carboxy-proxyl, HBTU, *i*Pr_2_EtN, DMA; *iv*. (a) LiOH⋅H_2_O, methanol/H_2_O, (b) glycine-HCl buffer, pH 2; *v*. (a) H_2_, Pd/C, methanol, (b) HCl. Abbreviations: 3Cx*p* = 3-carboxy-proxyl.

Syntheses of the two NBACs, CA-Px-1 and CA-Px-2, are outlined in Schemes 1 and 2.

Preparation of CA-Px-1 began with methyl esterification of commercially available *N*-(2-aminoethyl)glycine to give the protected linker, 1, which was isolated as the dihydrochloride salt. Condensing 1 with 3-carboxyl-proxyl gave protected linker-nitroxide 2. Condensation of 2 with CA yielded the protected conjugate, 3, which was deprotected with lithium hydroxide to yield the desired NBAC, 4 (CA-Px-1). Catalytic hydrogenation of 4 reduces the nitroxide to a hydroxylamine, which was isolated as the HCl salt, 5 (CA-Px-1H).

Preparation of CA-Px-2 began with condensation of CA with doubly-protected lysine linker, with Boc on the ε-amino and methyl on the α-carboxyl. The resulting protected CA-linker, 6, was treated with 6 *M* hydrochloric acid which removed the Boc group to give 7. Condensation of 7 with 3-carboxy-proxyl gave the protected conjugate, 8. Removing the methyl protective group of 8 with lithium hydroxide yielded the desired NBAC, 9 (CA-Px-2). Catalytic hydrogenation reduced the nitroxide to the corresponding hydroxylamine, which was isolated as the HCl salt, 10 (CA-Px-2H).

### 3.2. Bile acid transport kinetics

**Fig. 2.**
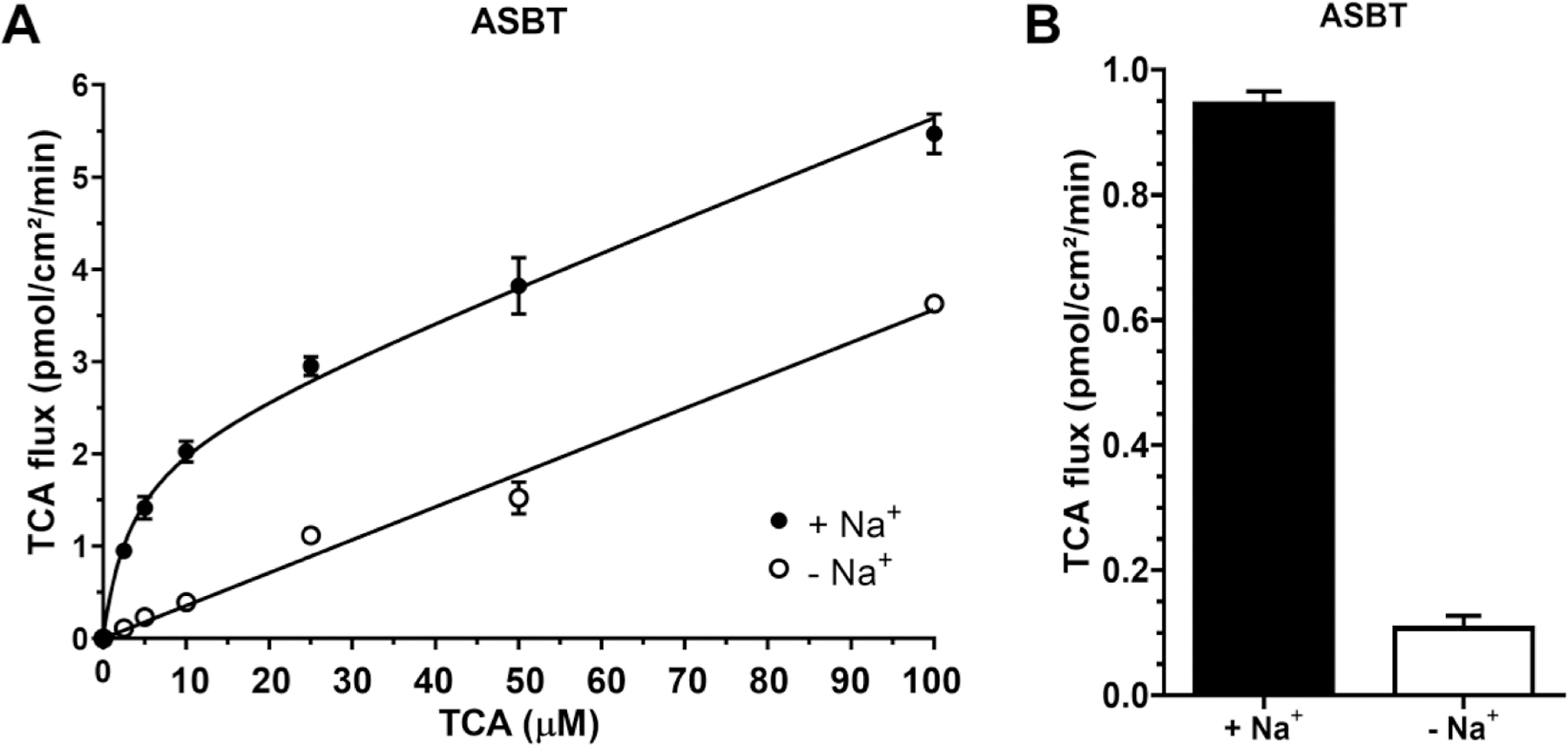
ASBT-mediated uptake of TCA. Data points represent uptake of TCA in the presence of Na^+^ (closed) and in the absence of Na^+^ (open). Each data point represents mean ± SEM (*n* = 3). (A) Concentration dependence of TCA (0–100 μM) uptake. (B) Effect of Na^+^ on TCA uptake (2.5 μM).

As shown in Fig. 2A, ASBT is a sodium-dependent transporter. In the presence of NaCl, TCA uptake displayed a Michaelis–Menten-like profile. Conversely, without NaCl, the passive transport of TCA across hASBT-MDCK cells exhibited a lower, linear profile. Regression analysis showed ASBT K_m_ = 3.35 ± 0.82 μM and V_max_ = 2.15 ± 0.11 pmol/cm^2^/min. Adding NaCl to 2.5 μM TCA increased TCA transport via ASBT approximately ten-fold (Fig. 2B).

**Fig. 3.**
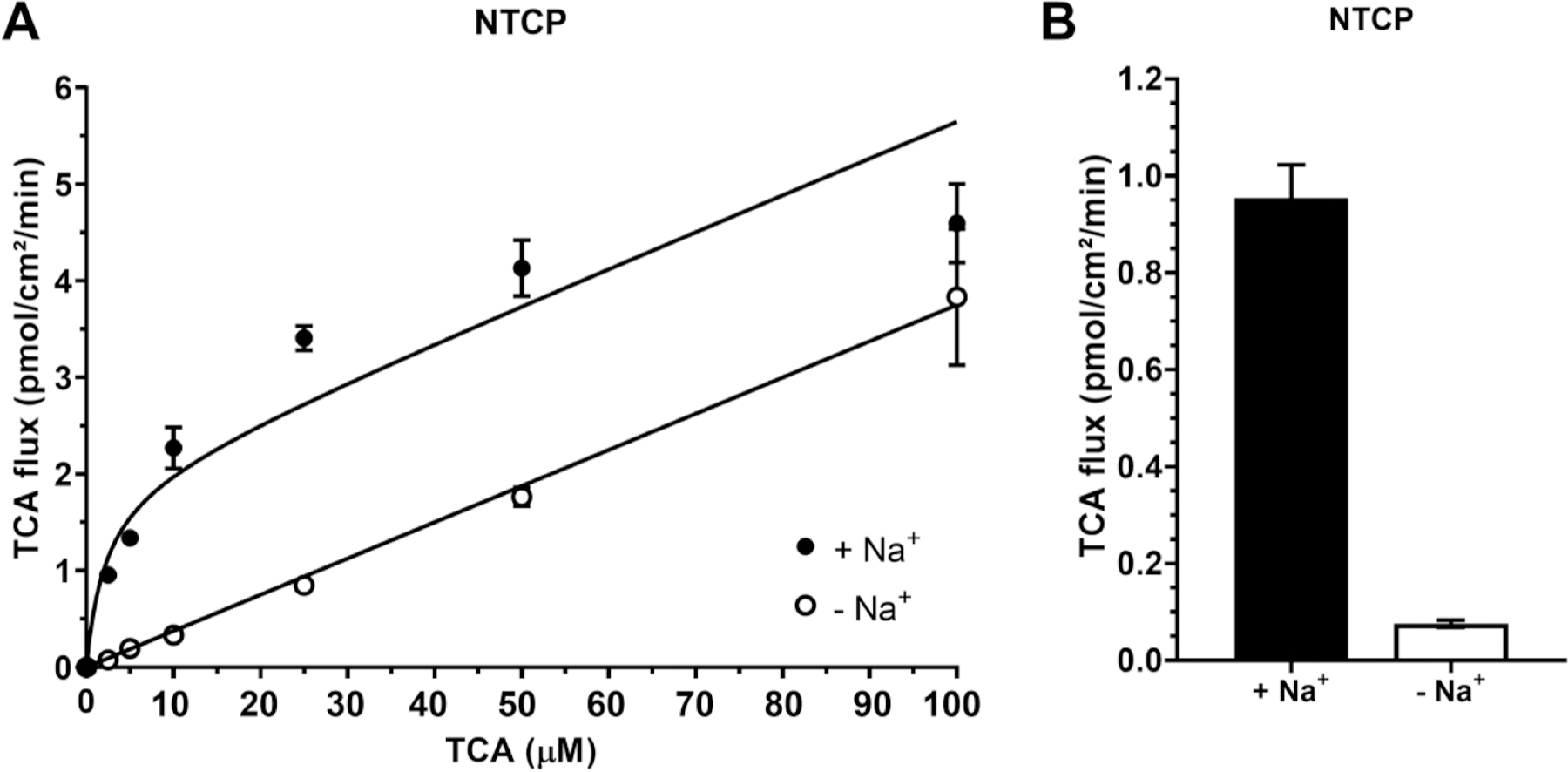
NTCP mediated uptake of TCA. Data points represent uptake of TCA in the presence of Na^+^ (closed) and in the absence of Na^+^ (open). Each data point represents mean ± SEM (*n* = 3). (A) Concentration dependence of TCA (0–100 μM) uptake. (B) Effect of Na^+^ on TCA uptake (2.5 μM).

As shown in Fig. 3A, NTCP is also a sodium-dependent transporter. In the presence of NaCl, TCA uptake showed a Michaelis–Menten-like profile. Conversely, without NaCl, the passive transport of TCA across hNTCP-HEK293 cells exhibited a lower, linear profile. Regression analysis showed NTCP K_m_ = 2.14 ± 1.55 μM and V_max_ = 1.94 ± 0.25 pmol/cm^2^/min. Adding NaCl to 2.5 μM TCA increased TCA transport via NTCP more than ten-fold (Fig. 3B).

### 3.3. Inhibition of ASBT and NTCP with GCA

**Fig. 4.**
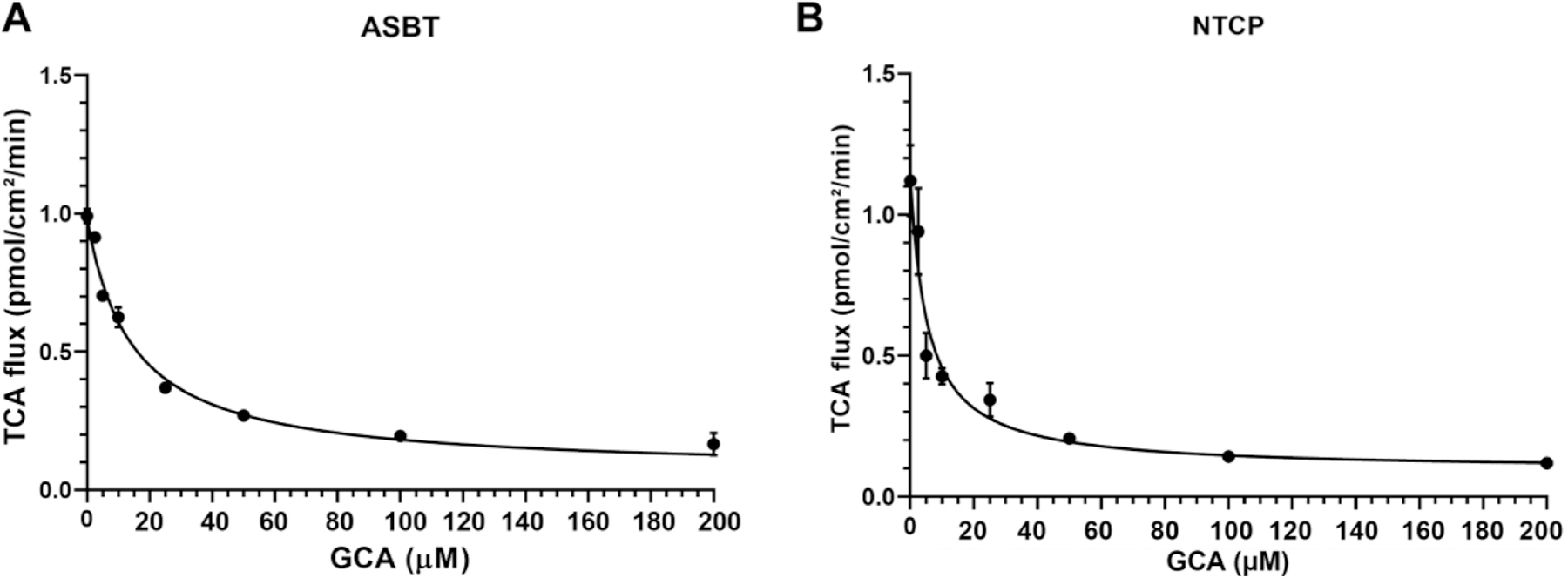
Inhibition of TCA uptake by glycocholic acid (0–200 μM) by (A) ASBT and (B) NTCP. Regression analysis showed ASBT K_i_ = 8.28 ± 0.57 μM and NTCP K_i_ = 2.47 ± 0.43 μM. Each data point represents mean ± SEM (*n* = 3).

GCA dose-dependently decreased active TCA transport into hASBT-MDCK and hNTCP-HEK293 cells (Fig. 4), consistent with competition for the same transporter [ASBT (A) and NTCP (B), respectively]. Regression analysis showed ASBT K_i_ = 8.28 ± 0.57 μM and NTCP K_i_ = 2.47 ± 0.43 μM. These findings show the assay’s ability to measure inhibition of *in vitro* transport by ASBT and NTCP.

### 3.4. Inhibition of ASBT and NTCP with NBACs

**Fig. 5.**
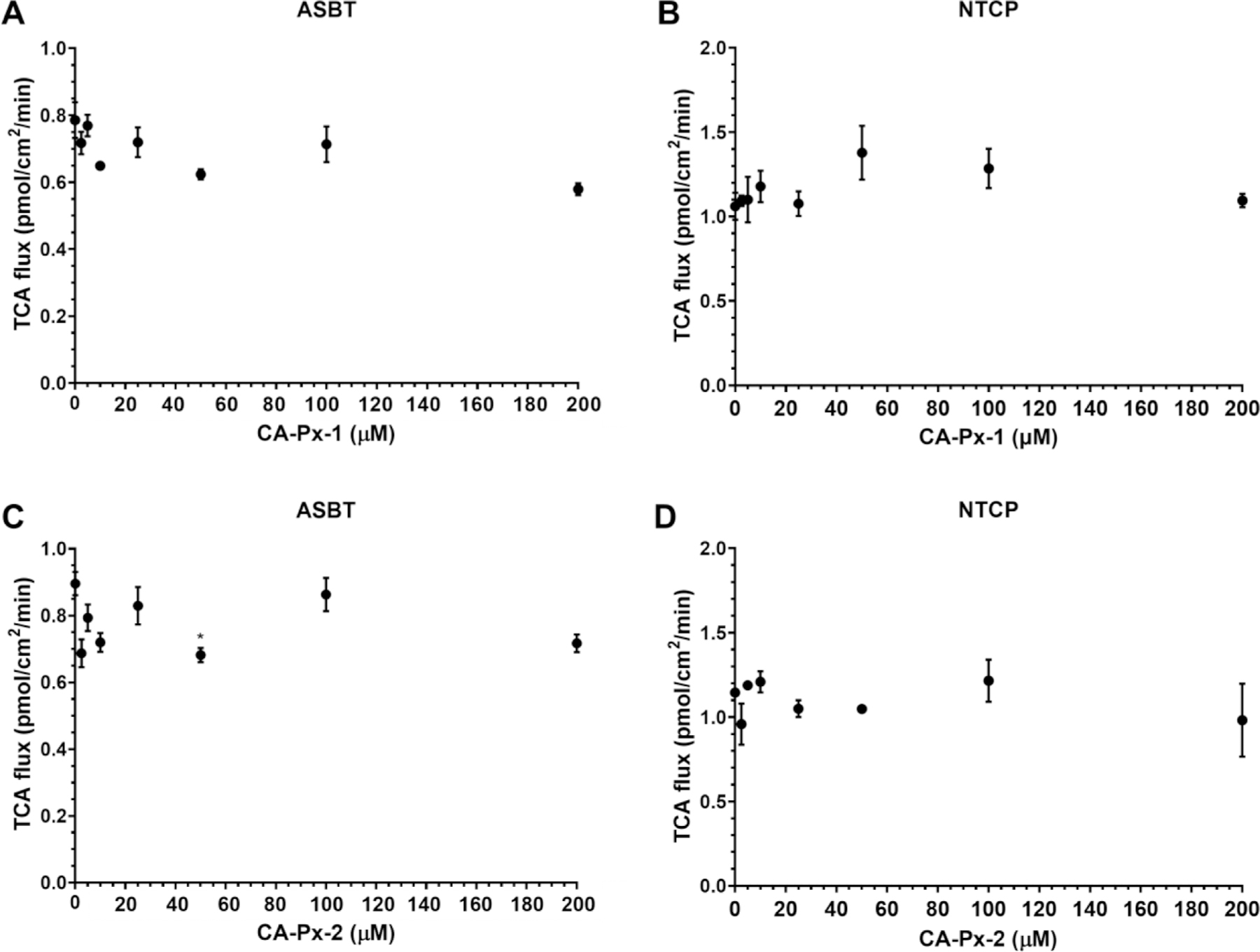
TCA uptake by (A, C) ASBT and (B, D) NTCP in the presence of (A, B) CA-Px-1 or (C, D) CA-Px-2. Each data point represents mean ± SEM (*n* = 3).

Transport and inhibition assays were used to characterize the newly devised NBACs, CA-Px-1 and CA-Px-2 (Fig. 5), on ^3^H-labeled TCA transport via ASBT (Fig. 5A and 5C, respectively) and via NTCP (Fig. 5B and 5D, respectively). In Fig. 5, CA-Px-1 and CA-Px-2 did not inhibit TCA transport via ASBT and NTCP. All p-values in Fig. 5A, B, and D were generally greater than critical p-value 0.007. In Fig. 5C, *p* = 0.007 for 50 μM CA-Px-2.

**Fig. 6.**
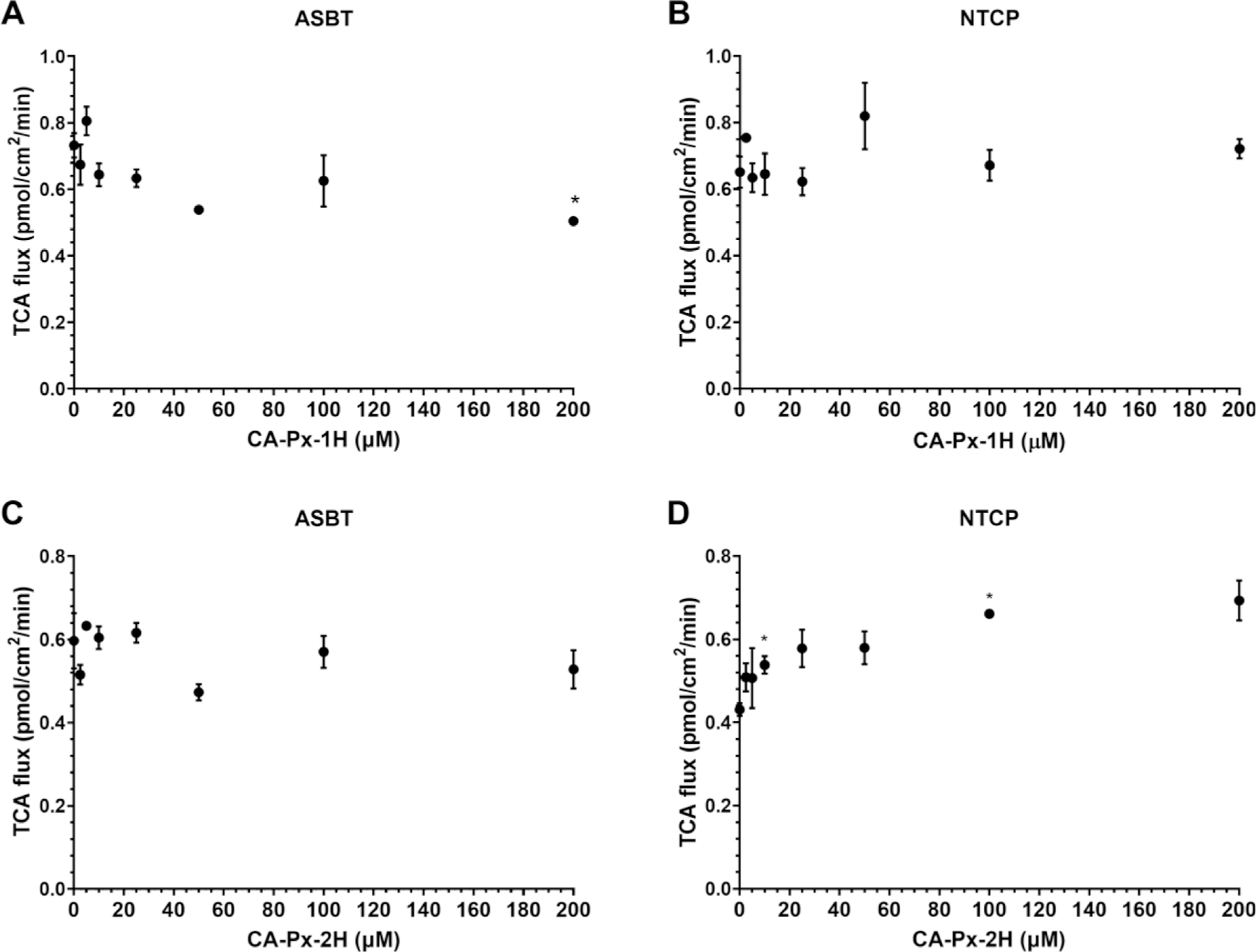
TCA uptake by (A, C) ASBT and (B, D) NTCP in the presence of (A, B) CA-Px-1H or (C, D) CA-Px-2H. Each data point represents mean ± SEM (*n* = 3).

To assess the impact of the nitroxide (N–O●) functional group on this observed lack of inhibition, the reduced forms of the NBACs, CA-Px-1H and CA-Px-2H (Fig. 6), were synthesized and subjected to ^3^H-labeled TCA inhibition studies of ASBT (Fig. 6A and 6C, respectively) and of NTCP (Fig. 6B and 6D, respectively). In Fig. 6, CA-Px-1H and CA-Px-2H did not inhibit TCA transport via ASBT and NTCP. All p-values in Fig. 6A were greater than 0.007 in all studies, except for 200 μM CA-Px-1H, where *p =* 0.005. All p-values in Fig. 6B and C were greater than 0.007. All p-values in Fig. 6E were greater than 0.007, except for 10 and 100 μM CA-Px-1H, where p-value equaled 0.007 and 0.002, respectively. Overall, it was concluded that neither CA-Px-1H nor CA-Px-2H inhibited ASBT nor NTCP.

## 4. Discussion

To assess bile acid transport within the enterohepatic circulation *in vivo*, we designed nitroxide-bile acid conjugates that were structurally similar to CA-sar-TFMA and CA-lys-TFMA, fluorinated conjugates of bile acids previously shown to undergo enterohepatic circulation (Felton et al., 2016; Metry et al., 2018; Raufman et al., 2019; Vivian et al., 2014a; Vivian et al., 2014b; Vivian et al., 2013). Fluorinated bile acid conjugates are potential MRI imaging agents to identify BAD and other disorders of bile acid synthesis and transport, but their translational value is limited by the requirement of specialized hardware and software to detect fluorine MRI signals (Metry et al., 2018; Raufman et al., 2019).

Literature on the structural requirements of hASBT and hNTCP substrates is limited (Grosser et al., 2021; Thongsri et al., 2021). Uncertainty regarding the molecular mechanisms underlying bile acid transport and the absence of high-resolution crystal structures of hASBT and hNTCP exacerbates the challenge of designing bile acid conjugates with transport capacity (Hu et al., 2011; Krause et al., 2018). Structural features essential for interaction with hASBT are summarized in Table 1. Notably, native bile acids such as chenodeoxycholic acid (CDCA) have been conjugated with a glutamic acid linker, to preserve the single negative charge around C-24, and structurally diverse moieties were attached to the linker. Increased polarity (e.g., esters and amine substituents on the benzene ring) has been shown to reduce ASBT affinity. Although dianions, cations, and zwitterions bind with high affinity, they are not substrates of ASBT (Balakrishnan et al., 2006a; Balakrishnan et al., 2006b). Similarly, using aniline conjugates of CDCA-glutamic acid, compound hydrophobicity promoted ASBT inhibition (Rais et al., 2010).

Some studies suggest that the substrate specificity of NTCP is much broader than ASBT (Anwer and Stieger, 2014; Claro da Silva et al., 2013; Dawson and Karpen, 2015). However, in a study using an *in vitro* 72-drug screen, we found NTCP was less permissive than ASBT to drug inhibition (Dong et al., 2013). Generally, NTCP substrates have a steroid scaffold, associated with the structural requirement for two hydrophobes and one hydrogen bond donor (Dong et al., 2015).

Sarcosine and lysine were used successfully as linkers in preparing the fluorinated bile acid conjugates demonstrated to undergo enterohepatic circulation (Felton et al., 2016; Metry et al., 2018; Raufman et al., 2019; Vivian et al., 2014a; Vivian et al., 2014b; Vivian et al., 2013). We therefore used the same amino acids to link CA to a nitroxide to generate the NBACs CA-Px-1 and CA-Px-2. The two nitroxide conjugates were tested for hASBT and hNTCP inhibition potential. We used reproducible *in vitro* models to detect and measure transport of the endogenous bile acid TCA by two key human bile acid transporters, intestinal ASBT and hepatic NTCP. Inhibition of TCA transport by glycocholic acid served as positive control. Surprisingly, the nitroxide conjugates did not inhibit TCA uptake by either transporter.

We asked whether the free radical moiety (N–O●) of the nitroxides could have impeded bile acid transport. Therefore, we reduced the nitroxides in CA-Px-1 and CA-Px-2 to yield the respective reduced (hydroxylamine) forms, CA-Px-1H and CA-Px-2H (Schemes 1 and 2). We anticipated that, if successfully translocated into the cell, the reduced forms would autooxidize intracellularly to the parent compounds, CA-Px-1 and CA-Px-2. The “masking” of the nitroxide moiety at various stages of enterohepatic circulation (e.g., in intestinal epithelial cells, hepatocytes, and the gallbladder) may be clinically useful. Unfortunately, however, CA-Px-1H and CA-Px-2H also did not inhibit TCA uptake via ASBT or NTCP. Therefore, the free radical nature of the conjugate is not the structural feature that prevents transport by ASBT and NTCP.

**Fig. 7.**
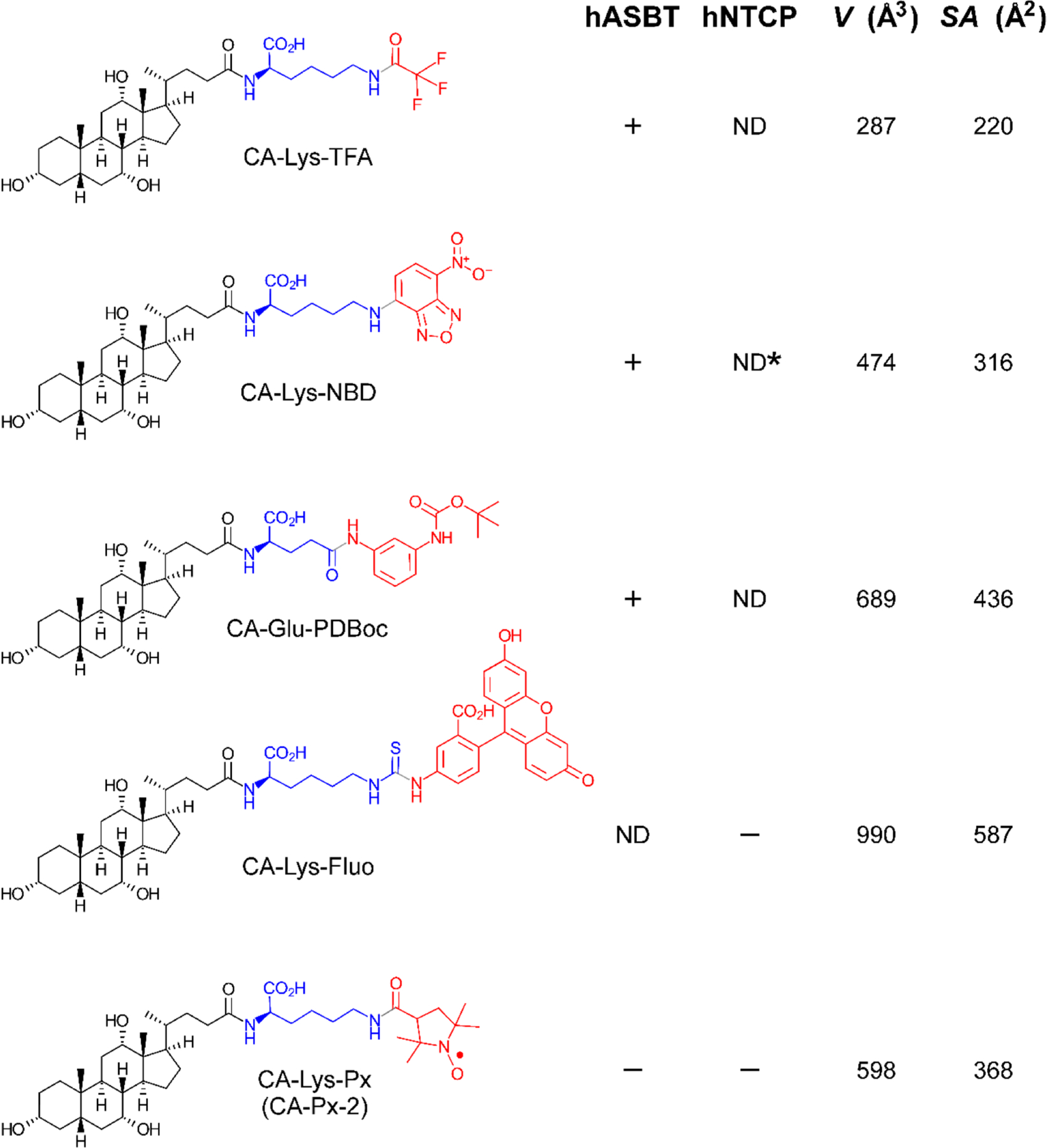
Comparison of cholic acid conjugates with structurally diverse species. For each species, the cholyl structure is shown in black, the linker in blue, and the conjugated moiety in red. The tripartite text labels indicate the bile acid (CA, cholic acid), the amino acid linker (Lys or Glu), and the conjugated species (TFA = trifluoroacetyl, NBD = nitrobenzoxadiazole fluorophore, PDBoc = 3-*t*-butoxycarbonylaminoaniline, Fluo = aminofluorescein, Px = proxyl nitroxide). The hASBT and hNTCP columns show whether the corresponding species is (+), or is not (−) a substrate of the human transporter (ND = not determined). The *V* and *SA* columns tabulate the molecular volume (in Å^3^) and surfaces area (in Å^2^) of the conjugated moiety; calculations were performed with HyperChem 8 (Hypercube, Inc.). Transporter properties were taken from the literature: [(Vivian et al., 2013), CA-Lys-TFA], [(Weinman et al., 1998), CA-Lys-NBD], [(Rais et al., 2010), CA-Glu-PDBoc], (de Waart et al., 2010) *CA-Lys-NBD is taken up by acutely isolated rat hepatocytes (Maglova et al., 1995).

An obvious possible reason for the nitroxide conjugates not being substrates of ASBT and NTCP is steric – the substituted pyrrolidine rings of the nitroxides may be too large to be accommodated by the transporters. This explanation seems unlikely, however, since structurally analogous cholyl conjugates with bulkier moieties are known to be substrates. As the comparison in Fig. 7 shows, the proxyl nitroxide conjugate is intermediate in size (in molecular volume and surface area) compared to known substrates of ASBT. We thus conclude that steric factors cannot rationalize our observations.

In conclusion, two nitroxide conjugates of bile acid and their reduced forms were synthesized. Surprisingly, none of these novel compounds exhibited bile acid transport properties, which makes them unsuitable as contrast agents for MRI. Findings from this study provide important insights into potential structural constraints on bile acid conjugates. In future work, we plan to explore conjugates where the nitroxide is attached to the steroid nucleus of the bile acid.

## Supplementary Material

1

## Figures and Tables

**Table 1:** Favorable structural elements for compound interactions with hASBT.

Structural Component	Advantage to hASBT	Refs.
Conjugation	Improves transport efficacy	Lack (1979)
Negative charge within C-24 region	Essential for interaction and transport	Lack (1979), Swaan et al. (1997)
14 Å or longer C-24 side chain	Translocation	Thongsri et al. (2021)
Large, hydrophobic moieties	Enhance binding	Thongsri et al. (2021)
Monoionic conjugate	Potent substrates	Thongsri et al. (2021)
Electron-donating and -withdrawing substituents	Potent substrates	Rais et al. (2010)

## Data Availability

Data will be made available upon request.

## Abbreviations:

ACN: acetonitrile
ASBT: apical sodium dependent bile acid transporter
BAD: bile acid diarrhea
BSEP: bile salt export pump
CA: cholic acid
CDCA: chenodeoxycholic acid
DIPEA: diisopropylethylamine
DMEM: Dulbecco’s modified eagle medium
DMF: *N,N*-dimethylformamide
DMSO: dimethyl sulfoxide
ESI: electrospray ionization
FBA: fluorinated bile acid
FBS: fetal bovine serum
FGF19: fibroblast growth factor 19
GCA: glycocholic acid
HBSS: Hanks’ balanced salt solution
HBTU: *N,N,N*′ *,N*′-tetramethyl-*O*-(1H-benzotriazol-1-yl)uronium hexafluorophosphate
HEK: human embryonic kidney
HRMS: high-resolution mass spectrometry
IBS-D: irritable bowel syndrome
MDCK: Madin-Darby canine kidney
MRI: magnetic resonance imaging
NBAC: nitroxide-bile acid conjugate
NMR: nuclear magnetic resonance
NTCP: Na^+^/taurocholate cotransporting polypeptide
ppm: parts per million
SFB: sodium-free buffer
TCA: taurocholic acid
TEA: tetraethylammonium
TLC: thin-layer chromatography
