# Supplementary material for: Novel nitroxide-bile acid conjugates inform substrate requirements for human bile acid transporters
Source: Eur J Pharm Sci. Author manuscript; Available in PMC 2023 Feb 8. (PMC9908032; doi:10.1016/j.ejps.2022.106335)
Supplement: 1 [file NIHMS1856921-supplement-1.pdf]

Supplementary Material

Metry *et al.*

1. ^{1}H-NMR spectrum of compound **1**
2. ^{1}H-NMR spectrum of compound **5** (CA-Px-1H)
3. ^{1}H-NMR spectrum of compound **6**
4. ^{1}H-NMR spectrum of compound **7**
5. ^{1}H-NMR spectrum of compound **10** (CA-Px-2H)

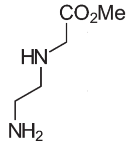

1

DMSO-d_6

INDEX	FREQUENCY	PPM	HEIGHT
1	3370.3	8.431	4.7
2	1626.9	4.070	81.1
3	1500.9	3.755	139.5
4	1353.8	3.387	23.8
5	1302.2	3.257	30.0
6	1296.7	3.244	29.5
7	1285.7	3.216	23.0
8	1280.2	3.203	24.3
9	1001.7	2.506	49.5
10	0.1	0.000	25.8

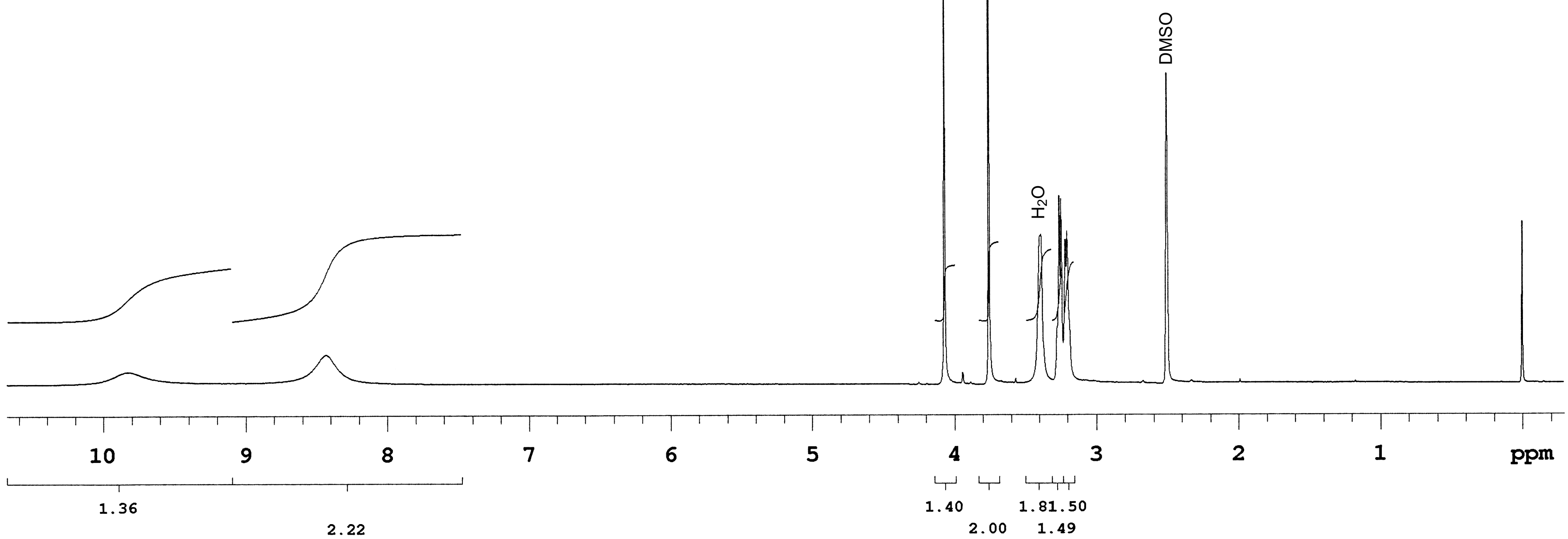

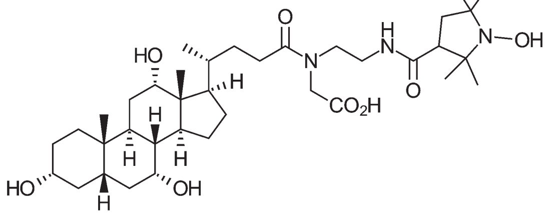

5 (CA-Px-1H)

D$_2$O

INDEX	FREQUENCY	PPM	HEIGHT
1	1906.7	4.770	697.5
2	831.3	2.079	101.7
3	634.7	1.588	123.7
4	628.8	1.573	136.2
5	596.5	1.492	115.3
6	543.0	1.358	100.1
7	402.9	1.008	50.1
8	364.0	0.910	113.7
9	286.9	0.718	84.0

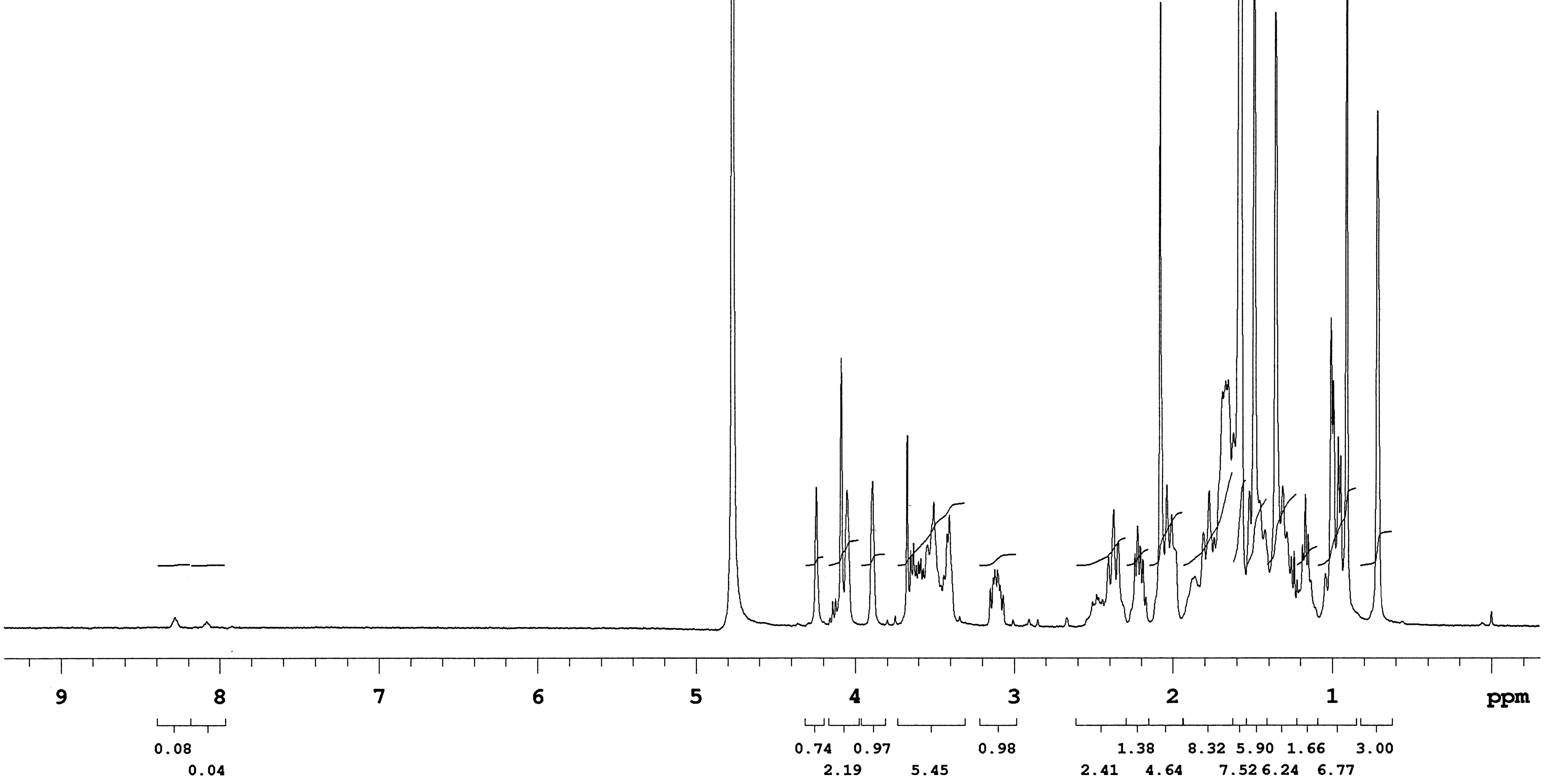

INDEX	FREQUENCY	PPM	HEIGHT
1	1438.2	3.598	39.1
2	1331.7	3.331	139.5
3	1000.0	2.501	71.9
4	795.7	1.991	16.6
5	546.9	1.368	110.2
6	508.5	1.272	19.0
7	502.3	1.256	32.3
8	496.8	1.243	24.7
9	469.4	1.174	10.9
10	375.5	0.939	18.8
11	369.3	0.924	17.1
12	323.1	0.808	34.8
13	233.1	0.583	32.7
14	-0.1	-0.000	39.7

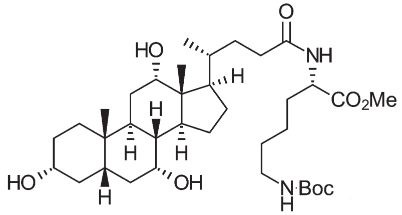

6

DMSO-d_6

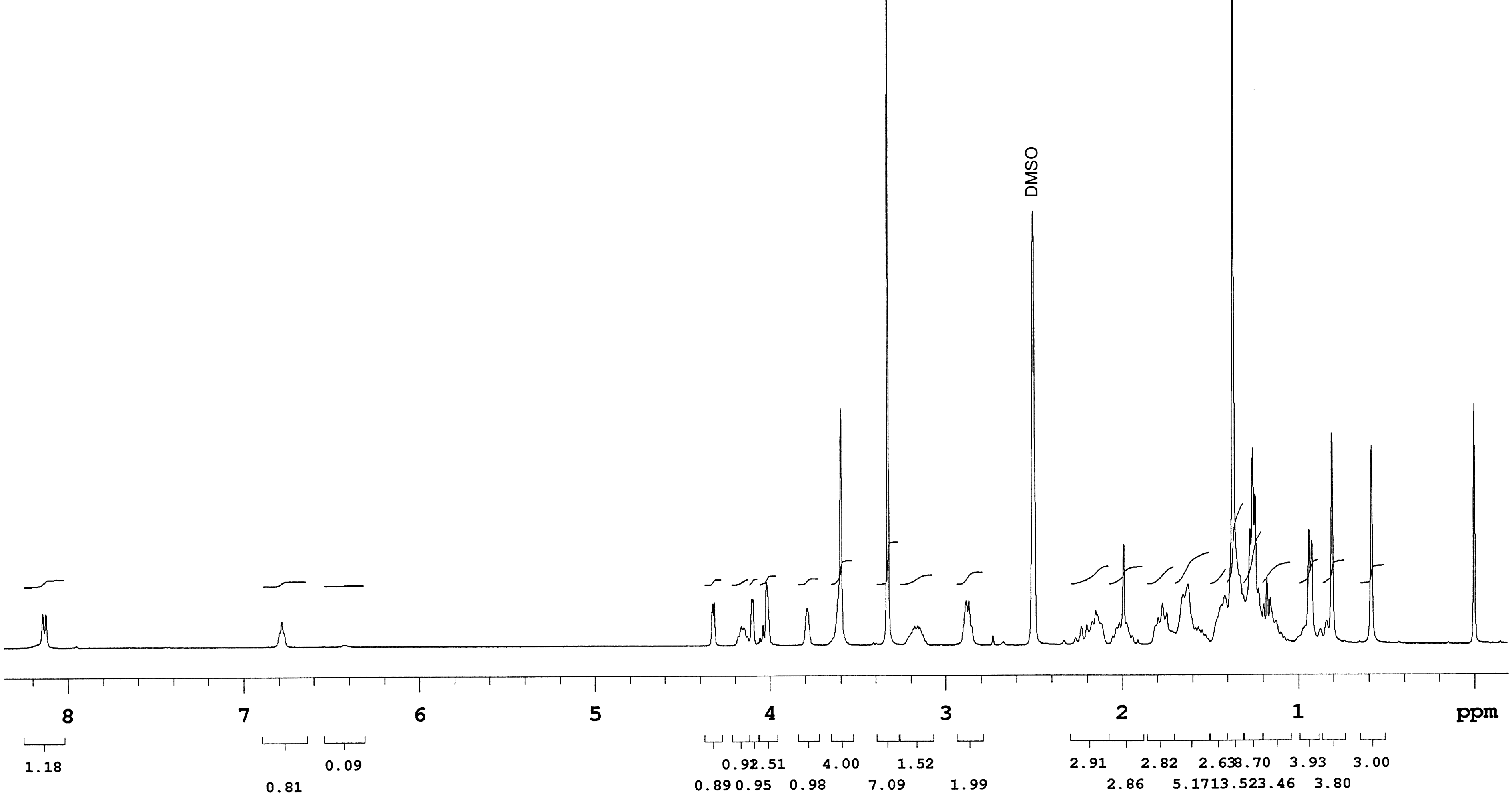

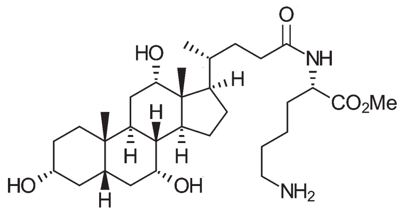

7

DMSO-d_6

INDEX	FREQUENCY	PPM	HEIGHT
1	3123.7	7.814	11.7
2	1514.9	3.790	10.7
3	1443.7	3.612	41.4
4	1426.5	3.568	139.5
5	1101.8	2.756	8.8
6	1096.3	2.742	9.2
7	1000.8	2.504	53.3
8	708.9	1.773	9.6
9	664.3	1.662	12.7
10	650.3	1.627	12.9
11	638.5	1.597	11.4
12	615.0	1.539	9.3
13	567.3	1.419	11.6
14	554.8	1.388	13.1
15	541.5	1.355	18.0
16	532.1	1.331	14.0
17	519.6	1.300	26.9
18	513.3	1.284	30.0
19	507.8	1.270	34.1
20	501.6	1.255	29.7
21	443.7	1.110	12.7
22	377.9	0.945	27.7
23	371.7	0.930	26.0
24	324.0	0.810	45.4
25	234.8	0.587	44.8
26	0.0	0.000	30.9

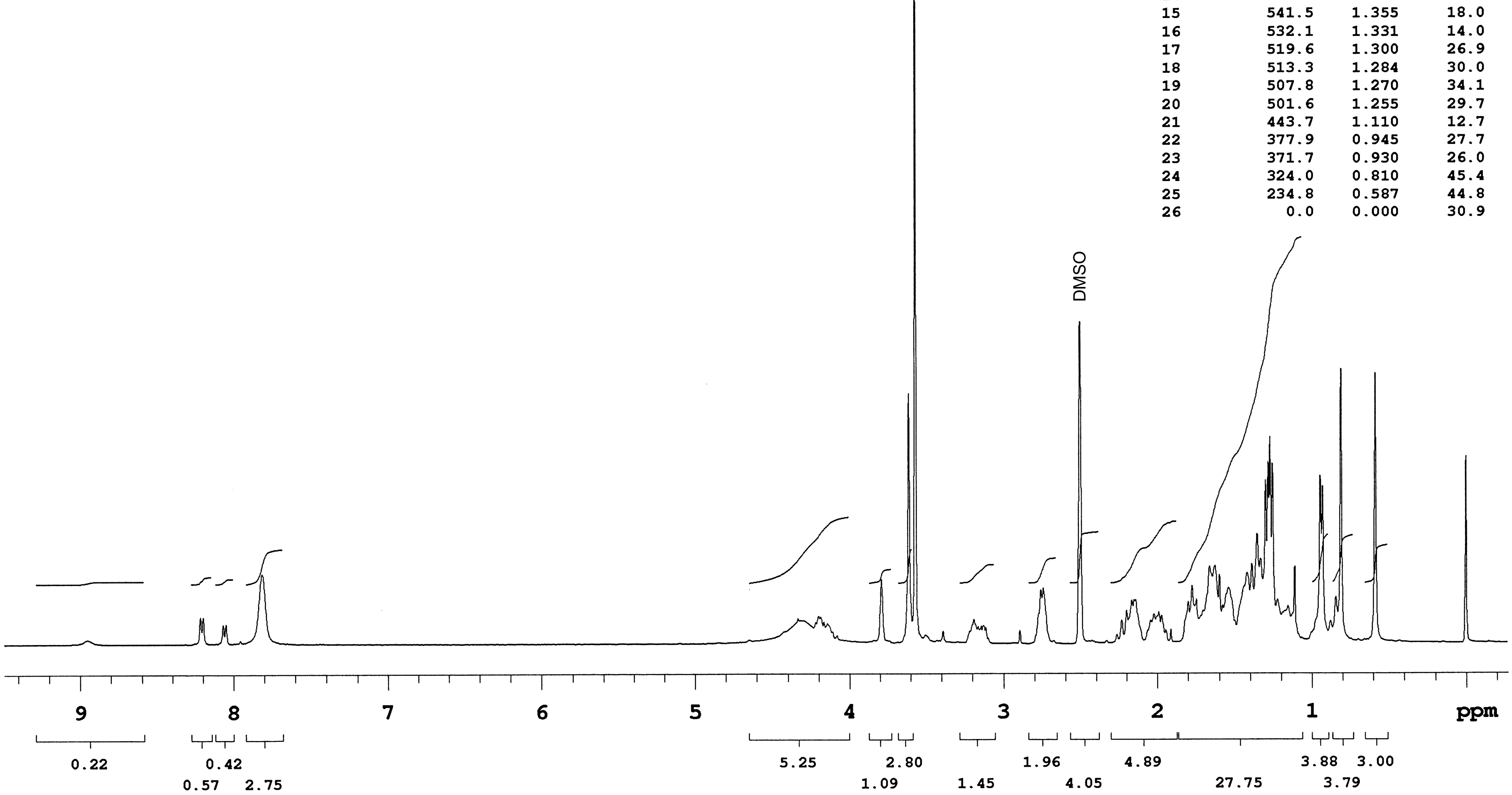

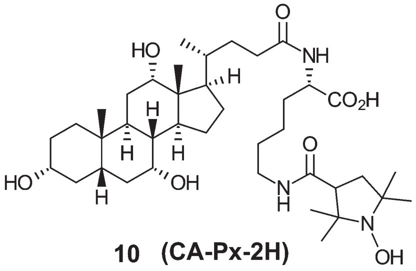

10 (CA-Px-2H)

D_2O

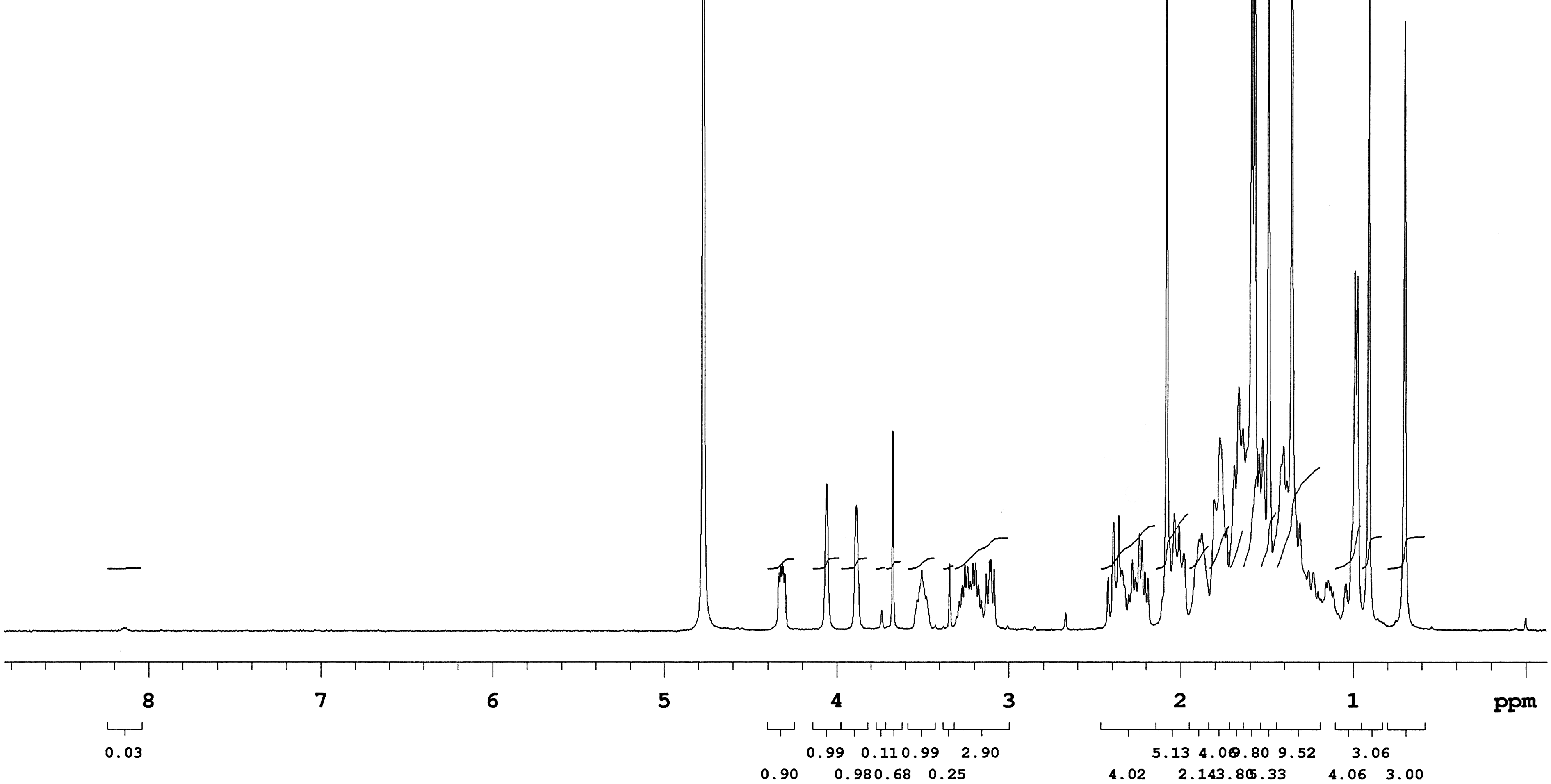

INDEX	FREQUENCY	PPM	HEIGHT
1	1907.3	4.771	346.5
2	831.1	2.079	107.6
3	636.0	1.591	139.5
4	628.6	1.573	131.0
5	596.4	1.492	121.7
6	542.8	1.358	121.5
7	395.4	0.989	58.2
8	389.5	0.974	57.3
9	363.1	0.908	105.1
10	279.5	0.699	98.6
